# Editorial: Efficacy of small muscle mass exercise training to promote health

**DOI:** 10.3389/fspor.2023.1217119

**Published:** 2023-05-26

**Authors:** Angela V. Bisconti, Stefano Longo, Ryan Broxterman, Jason R. Gifford, Emiliano Cè

**Affiliations:** ^1^ Department of Internal Medicine, University of Utah, Salt Lake City, UT, United States; ^2^ Deptartment of Biomedical Sciences for Health, University of Milan, Milan, Italy; ^3^ Geriatric Research, Education, and Clinical Center, Veterans Affairs Medical Center, Salt Lake City, UT, United States; ^4^ Department of Exercise Sciences, Brigham Young University, Provo, UT, United States

**Keywords:** small muscle mass, single-leg cycling, fidget factor, blood flow restriction exercise, isometric hand grip, unilateral training

**Editorial on the Research Topic** Efficacy of small muscle mass exercise training to promote health

This research topic seeks to highlight the use of small muscle mass exercise training (active or passive), instead of the whole-body exercise training protocol, as a novel and non-pharmacological approach for maintaining and improving immediate and long-term health, especially in individuals with limited mobility or where the more traditional exercise approaches are not viable.

According to the Centers for Disease Control and Prevention (CDC), regular physical activity can be considered a powerful tool to improve and maintain health, moving more and sitting less have tremendous benefits for everyone's health (i.e., brain and heart health, cancer prevention, healthy weight, bone strength, balance, and coordination), despite age, sex, or current fitness level (1).

Interestingly, as physical activity is defined as any bodily movement produced by skeletal muscles that require energy expenditure, the exercise, instead, as a subcategory of physical activity, is planned, structured, repetitive, and purposefully focused on the improvement or maintenance of one or more components of physical fitness (2).

Despite these differences in definition between exercise and physical activity exists, a recent review outlined that the Fidget Factor, i.e., those impulses that precipitate movements that range from barely perceptible fidgets (e.g., the tap of a finger) to a larger motion (e.g., the crossing of legs) may have an important impact on health. Indeed, in a modern chair-based lifestyle, Fidget Factors are suppressed, meaning that people's natural tendency to move is overridden, determining excess sitting and decline in the physical and mental health state leading to earlier death from sedentariness. Quite the reverse, Fidget Factor–permissive environments enable people to reverse sedentariness leading people to be healthier, happier, smarter, and more productive (Levine).

More on the physical exercise side, a recent systematic review investigated the use of isometric handgrip as a relevant small muscles exercise model to possibly improve cognitive performance in healthy adults (Zhu et al.). Despite the inconclusive results in response to a single acute isometric handgrip bout, the present work points toward a positive effect on cognitive function in response to chronic exercise (i.e., training protocol) (Zhu et al.). Specifically, it seems that 8 weeks of isometric handgrip could significantly improve the cognition processing speed along with blood pressure and handgrip strength, the latter both associated with cognitive function, especially in older adults. Although this observation is promising, especially since positive results were observed in healthy adults where cognitive function is mostly preserved, it should also be treated cautiously due to the relatively small number of studies available. Anyway, this review is a call for future large-scale clinical trials to fully elucidate whether chronic handgrip can be an effective intervention strategy to improve or maintain measures of cognitive performance, especially in populations more prone to develop cognitive impairment.

Remarkably, several studies over the years investigate the use of single-leg unilateral training (i.e., lunge squats, rear foot elevation split-leg squats, single-leg drop jumps, etc.), as a complementary and alternative method to the most common bilateral training (i.e., squat, deadlift, bench press, etc.). A meta-analysis systematically and objectively evaluates the exact effects of unilateral and bilateral exercises on different modulators of athletes' physical performance and found that unilateral training has a sport specificity, especially for sports with unilateral limb dominant force, and, therefore, has a more significant effect on jumping ability and maximum strength for unilateral power generation patterns (Zhang et al.). Additionally, unilateral training galvanizes a phenomenon known as cross migration phenomenon for which the unilateral activation of one muscle could improve muscle strength and neural activity of the contralateral homologous muscle, without any significant increase in the cross-sectional area. This phenomenon assumes that the adaptation and regulation of the neuromuscular system occur in the cerebral cortex and spinal cord, which is weakly influenced by myogenic factors.

Similar to unilateral training, also single-leg cycling (SLC) has been widely investigated as an alternative exercise modality to promote and maintain health. A recent review summarized data on the acute physiological responses and long-term adaptations to SLC for populations ranging from trained endurance athletes to individuals living with chronic disease (Heidorn et al.). In detail, acute responses for SLC in a healthy population included: 30%–90% greater blood flow to the active leg despite similar heart rate and blood pressure when the workload is matched between single and double-leg cycling; Greater limb-specific work rate, and oxygen consumption, accomplished with reduced cardiovascular demand; enhanced limb-specific substrate utilization, and possibly offset oxygen-related decreases in training performance at altitude. In the disease population, the major acute responses included increasing exercise tolerance, such as time to exhaustion, and limb-specific work rate, and improving peripheral adaptation, such as blood flow. As far as chronic training adaptations following SLC concerns, the healthy population reported that a training period of ≥6 weeks with a frequency of ≥2 sessions/week determines improvements in single-leg oxygen uptake, higher citrate synthase activity, greater arteriovenous oxygen difference, better substrate energy utilization, and a decrease of the main cardiovascular risk factors such as systolic blood pressure, and blood lipids. Significant chronic training adaptations were also found in disease populations, indeed SLC training improved oxygen uptake and kinetics, peripheral vascular function, and functional performance such as walking, and the observed benefits may be greater than those following double-leg cycling training in various clinical populations. Anyway, the main limitation of this specific modality is the need for slight ergometer or pedaling (emphasis) modifications. In the future additional research is needed to better understand how to incorporate SLC into weekly exercise/training routines.

Last but not least, a meta-analysis found that the implementation of blood flow restriction training resulted in a significant improvement in lower limb muscle strength (Yang et al.). The optimal conditions for blood flow restriction training, as determined by the meta-analysis, were as follows: a training load of 30% or less of one repetition maximum, a training duration exceeding 4 weeks, a training frequency exceeding 3 sessions per week, and a maximum cuff pressure of no more than 200 mmHg.

In summary, while much is still needed to unfold, primary peripheral adaptations as consequences of small muscle mass training could potentially have widespread benefits on cardiovascular risk and exercise tolerance locally and globally. With this preliminary evidence in mind, it's not unreasonable to state that small-muscle exercise mass training, similar to whole-body exercise training, may be considered a safe tool to promote and maintain immediate and long-term health, especially in individuals with reduced mobility or when traditional exercise methods are not feasible.
